# Oral supplementation with piceatannol improves skin hydration and reduces wrinkle severity: a randomized, double-blind, placebo-controlled trial

**DOI:** 10.3389/fnut.2026.1765478

**Published:** 2026-04-28

**Authors:** Yosuke Seto, Motoki Tsukiashi, Takumi Yamaguchi, Shinpei Kawakami, Hiroko Maruki-Uchida, Eisaku Nishimura, Sadao Mori, Mariko Ito, Kumi Jin, Kazuhisa Maeda, Naoki Iemoto

**Affiliations:** 1 R&D Institute, Morinaga & Co., Ltd., Yokohama, Japan; 2 Nihonbashi Ueda Clinic, Tokyo, Japan; 3 Tokyo University of Technology, Tokyo, Japan

**Keywords:** aging, clinical trial, piceatannol, polyphenol, SIRT1, skin, skin hydration, wrinkle

## Abstract

**Background/objectives:**

The skin is the largest organ of the human body and undergoes age-related changes, such as reduced hydration, loss of elasticity, and wrinkle formation, which can affect appearance and quality of life. Piceatannol has antioxidant and anti-inflammatory properties and has attracted attention as a functional ingredient for maintaining skin health. The current study aimed to evaluate the effects of piceatannol intake on skin hydration and the severity of wrinkles.

**Methods:**

In a randomized, double-blind, placebo-controlled trial, healthy Japanese women aged 30–59 years were made to ingest a drink containing 10 mg of piceatannol derived from passion fruit seeds, or a matching placebo, once daily for 8 weeks. Stratum corneum hydration, and wrinkle grades were assessed at baseline and at the end of the treatment.

**Results:**

Eighty-six participants were enrolled in the trial and 82 participants were included in the analysis. The piceatannol group exhibited a statistically significant increase in facial stratum corneum hydration compared to the placebo group. In addition, wrinkle grades showed that wrinkles improved significantly in the piceatannol group compared to in the placebo.

**Conclusion:**

Intake of piceatannol could serve as an effective dietary intervention for improving skin hydration and wrinkles.

**Clinical trial registration:**

https://center6.umin.ac.jp/cgi-open-bin/icdr_e/ctr_view.cgi?recptno=R000064440, identifier UMIN000056580.

## 1 Introduction

The skin is the largest organ of the body; it not only covers its surface but also serves as a physiological interface between the external environment and the internal environment. It is composed of three main layers—the epidermis, dermis, and subcutaneous tissue—which together perform essential roles in barrier protection, moisture retention, sensory perception, and thermoregulation (1). The stratum corneum, the epidermis’s outermost layer, consists of corneocytes, intercellular lipids (such as ceramides, cholesterol, and fatty acids), and natural moisturizing factors (NMFs), which together organize into a lamellar structure that retains water and defends against the invasion of foreign substances, thereby performing the barrier function (2). The dermis contains abundant fibrous proteins (collagen and elastin) and extracellular matrix components (notably hyaluronic acid), which are critical for skin firmness, elasticity, and hydration (2). When elasticity and hydration become impaired, barrier dysfunction, inflammation, dryness, and wrinkle formation can follow. Thus, preserving stratum corneum hydration and elasticity is vital for skin health.

During the aging process, the skin undergoes modifications in its structure and function, one of which is reduced hydration in the stratum corneum (3), decreased elasticity and firmness due to loss of collagen and hyaluronic acid, and formation of wrinkles and sagging (4). In addition, the skin accumulates damage from external factors such as ultraviolet-induced photoaging (5). Such alterations can negatively affect the appearance and self-image, potentially resulting in diminished quality of life (QOL) and reduced social activity (6). In a super-aging society, extending healthy life expectancy and maintaining QOL are of increasing importance; interventions that prevent moisture loss and wrinkle or sag formation, thereby preserving skin structure, may contribute to an overall well-being.

Against this backdrop, various strategies for preserving skin health and delaying aging have been proposed, with particular attention to “skin health from within” through dietary ingredients and supplements. Collagen peptides (7), hyaluronic acid (8), and polyphenols (9) are widely used as health food ingredients and have demonstrated efficacy in improving skin hydration, elasticity, and wrinkle appearance. Polyphenols, in particular, possess strong antioxidant and anti-inflammatory properties that mitigate ultraviolet damage and suppress skin aging processes (10). Piceatannol (PIC), a stilbene compound structurally related to the anti-aging agent resveratrol, has been reported to be highly concentrated in passion fruit seeds (11). Compared to resveratrol, PIC exhibits higher bioavailability in its unmodified form (12) and has been shown to exert diverse physiological effects, including suppression of postprandial blood glucose (13, 14), vasodilation (15), protection against mitochondrial dysfunction (16), suppression of fat accumulation, and activation of hormone-sensitive lipase (17, 18). Furthermore, *in vitro* and human studies have demonstrated that PIC upregulates the expression of the longevity- and anti-aging-related gene Sirtuin 1 (SIRT1), highlighting its potential in the field of anti-aging research (19, 20).

At the cellular level, PIC has been shown to suppress UV-induced reactive oxygen species generation, promote collagen synthesis, inhibit its degradation in dermal fibroblasts, and also inhibit melanogenesis (11, 21). Research has also indicated potential applications of PIC in skincare products (22, 23). In clinical trial, ingestion of PIC-containing foods was found to be associated with improvements in stratum corneum hydration and skin elasticity (24, 25); however, the specific effects on wrinkle formation—an age-related change that becomes increasingly apparent—remain largely unclarified. Therefore, the present study was designed to evaluate the impact of PIC derived from passion fruit seeds on both wrinkle severity, and skin hydration in individuals concerned about skin dryness.

## 2 Materials and methods

### 2.1 Clinical trial design

This clinical trial was conducted in accordance with the ethical principles of the Declaration of Helsinki (revised in 2013) and Ethical Guidelines for Life Science and Medical Research Involving Human Subjects. Ethical approval was obtained from the Clinical Research Review Center (General Incorporated Association; approval no. CrrC24-041) on November 14, 2024. Prior to enrollment, all participants received detailed information about the study’s objectives, procedures, and potential risks and provided written informed consent. Participant recruitment and management were handled by 701 Research, Inc. (Tokyo, Japan), and measurements were conducted at the Oak Otsuka Bld. Data collection, and management were overseen by an independent researcher to ensure data integrity, and compliance with the study protocol. The trial ran from January to April 2025 and was registered with the UMIN Clinical Trials Registry (UMIN ID: UMIN000056580).

### 2.2 Participants

The current study selected only the participants who met the following inclusion criteria but not any of the exclusion criteria.

Inclusion criteria: (1) Japanese women aged ≥ 30 but < 60 years at the time of informed consent; (2) healthy individuals with no chronic disease, including skin diseases; (3) individuals who are concerned about skin dryness; (4) individuals who voluntarily agree in writing to participate in the study; (5) individuals who are able to visit the study site on designated test days and undergo examinations; and (6) individuals judged appropriate for participation in this study by the principal investigator.

Exclusion criteria: (1) individuals who are currently under pharmacological treatment for any diseases; (2) individuals who currently have or are being treated for skin diseases; (3) individuals who have wounds or inflammation in the evaluation area; (4) individuals with a current or past history of serious disorders of the liver, kidneys, heart, lungs, or blood; (5) individuals with comorbidity or past gastrointestinal diseases; (6) individuals with alcohol dependence or psychiatric disorders; (7) individuals who used or applied medications for the treatment of diseases within the past month; (8) individuals who may not be able to control seasonal allergy symptoms with their usual medications during the study period; (9) individuals with food allergies; (10) individuals with serious anemia; (11) individuals with a smoking habit; (12) individuals who are pregnant, breastfeeding, possibly pregnant, or planning to become pregnant during the study period; (13) individuals who may change their lifestyle during the study period; (14) individuals who cannot refrain from intentional sun exposure, such as tanning, during the study period; (15) individuals who currently or within the past 3 months have a habitual intake of functional foods or supplements containing ingredients in the test food, or plan to take them during the study period; (16) individuals who currently or within the past 3 months have a habitual intake of, or plan to take during the study period, functional foods or supplements that claim to improve skin condition; (17) individuals who currently or within the past 3 months have a habitual use (oral or topical) of medicines, quasi-drugs, or cosmetics that may affect the study results; (18) individuals who have conducted hormone replacement therapy within the past 6 months; (19) individuals currently (or within the past 6 months) undergoing special facial care at external institutions; (20) individuals who have received cosmetic procedures or cosmetic medical treatments within the past 6 months; (21) individuals who have received inpatient treatment within the past 6 months; (22) individuals who participated in another clinical trial within 1 month before obtaining consent for this study, or plan to participate in another clinical trial after providing consent for this study; and (23) individuals judged inappropriate for this study by the principal investigator.

### 2.3 Test food

The test food was a 125 mL drink containing PIC, while the placebo drink comprised an identical 125 mL drink formulated the same way but without PIC. Participants consumed one pack of their assigned drink each day for 8 weeks, providing the PIC group with 10 mg of piceatannol per day.

### 2.4 Experimental protocol

This was a randomized, double-blind, placebo-controlled, parallel-group comparison trial designed to investigate the effects of the test food. A total of 124 participants were initially recruited, and they all underwent baseline screening measurements.

At the first clinic visit, participants received an explanation of the study and underwent a medical interview to confirm eligibility according to the inclusion and exclusion criteria. Baseline measurements and evaluations relevant to the study endpoints were performed during this visit. Based on baseline stratum corneum water content, participants with relatively lower hydration levels were prioritized for inclusion to facilitate the detection of intervention-related changes. The sample size was determined based on the results of a previous exploratory study evaluating the effects of this test food on skin hydration. An effect size of 0.64 was calculated from the previous data, and with a power of 0.8 and a significance level of 0.05, the required number of participants was calculated to be 40. Taking possible dropouts into consideration, the final sample size was set at 43.

The 86 participants were then randomly allocated to two groups using stratified block randomization (block size = 2) based on age and baseline stratum corneum water content (*n* = 43 each), ensuring no significant difference between the groups at baseline. Subsequently, an independent controller assigned each group to either the test or placebo food (test food: PIC group, placebo food: placebo group). The assigned test food information was kept confidential until analysis was completed. The participants consumed one pack per day of either the test or placebo food between dinner and bedtime for eight consecutive weeks.

During the study, they were instructed not to make any substantial change in lifestyle, such as excessive exercise or overeating. Any change in physical condition or missed intake of the test or placebo food was recorded. At the end of the 8-week intake period, endpoint measurements and evaluations were repeated during the final clinic visit. Evaluations were conducted at baseline and after 8 weeks of intake, as the study focused on the effects of sustained PIC consumption rather than its temporal kinetics. Details regarding measurement methods are described in the subsequent sections.

### 2.5 Skin hydration

On the measurement day, after washing and rinsing their faces, participants were acclimated in the humidity-controlled room (21 ± 1 °C and 50 ± 10% humidity) for at least 20 min. After acclimation, skin hydration was evaluated using two devices on both the left and right cheeks. The SKICON-200EX (Yayoi Co., Ltd., Tokyo, Japan) assessed stratum corneum water content by measuring high-frequency (1.25 MHz) conductance. The Corneometer CM 825 (Courage+Khazaka Electronic, GmbH, Cologne, Germany) measured stratum corneum water content using a capacitance method. Five recordings were obtained for each area, and the average was calculated.

### 2.6 Wrinkles

Wrinkle grades were assessed visually using the grading system defined by the Japanese Cosmetic Science Society (JCSS) (26). Wrinkle grades were targeted the lateral canthal area of the eye to evaluate crow’s feet. Grading was conducted following the official guidelines by the expert evaluators as described in the guidelines to classify wrinkles into eight grades (0–7) with quarter-point (0.25) increments.

### 2.7 Statistical analysis

Background data of the participants are presented as the means ± standard deviation of the mean (SD) while other data are presented as means ± standard error of the mean (SE). For all statistical analyses, EZR (ver. 1.68) was used (27). For each endpoint, analysis of covariance (ANCOVA) was performed, with the post-intervention value as the dependent variable, group (PIC group or the placebo group) as a fixed factor, and baseline value as a covariate. Statistical significance was set at *p* < 0.05 (two-sided). Effect sizes were calculated and are provided as Cohen’s d.

## 3 Result

### 3.1 Participants

**Figure 1:**
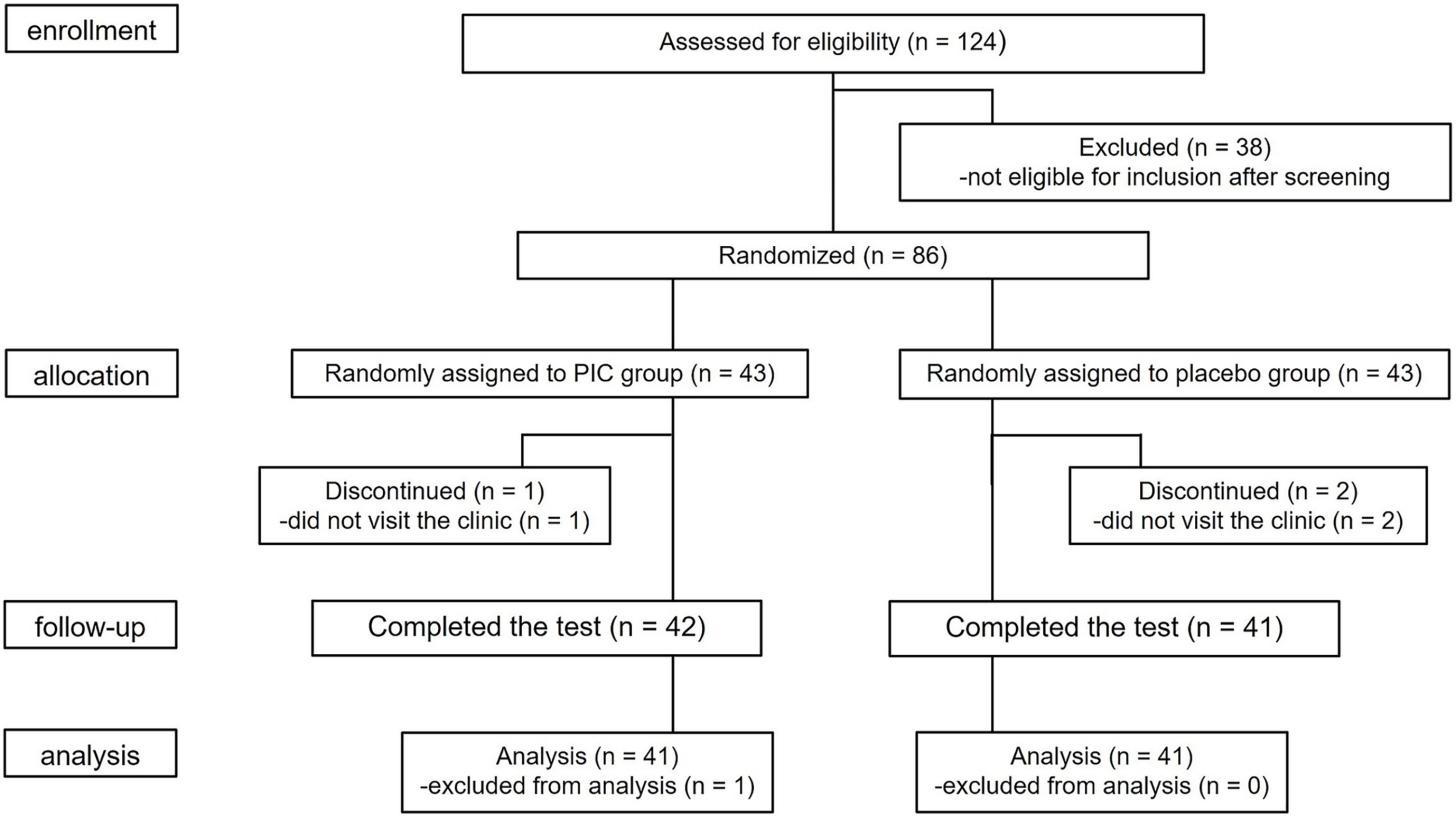
CONSORT flow diagram of the participants in this study. PIC, piceatannol.

The study flowchart is presented in Figure 1. A total of 124 volunteers were recruited initially. On the basis of baseline measurements, those with lower stratum corneum hydration were given priority, and a total of 86 participants were ultimately enrolled. The 86 individuals were then randomized in equal numbers—43 each—to receive either the test food or a placebo. During the study period, three participants could not complete the study due to personal reasons, resulting in a final of 83 participants who finished the trial. In addition, one participant developed a skin disorder during the study period, and although it was judged by the study physician to be unrelated to the test food, the participant’s data were excluded from the analysis. Consequently, data from 82 participants (41 in each group) were included in the efficacy analysis. The participants’ baseline characteristics are presented in Table 1. No significant difference was found between the groups in terms of age, hydration of the stratum corneum, or wrinkle grades.

**Table 1:** Baseline characteristics of the study participants.

Variable	PIC (*n* = 41) Mean ± SD	Placebo (*n* = 41) Mean ± SD	*p*-value
Age (year)	49.5 ± 6.7	49.9 ± 7.0	0.773
Height (cm)	159.2 ± 5.9	158.2 ± 5.6	0.415
Weight (kg)	56.9 ± 10.2	54.2 ± 9.7	0.228

SD, standard deviation.

### 3.2 Skin hydration

The results for stratum corneum water content are summarized in Table 2. After 8 weeks of consumption, the PIC group showed significantly higher stratum corneum water content at the facial site than the placebo group, as assessed by two measurement devices. The post-intervention value obtained with the SKICON-200EX was significantly higher in the PIC group than in the placebo group (*p* = 0.001). Similarly, with the Corneometer CM825, the PIC group showed a significantly higher value than the placebo group (*p* = 0.044). To visually illustrate the effects of PIC intake, graphical representations of stratum corneum water content are provided in the Supplementary Figure S1.

**Table 2:** Measurement of skin hydration using SKICON-200EX, and Corneometer CM825.

Evaluation parameter	Group	Baseline	Week 8	Change from baseline	Group differences	*p*-value	Cohen’s *d*
		Mean ± SE [95% CI]	Mean ± SE [95% CI]	Mean ± SE	Mean ± SE		
SKICON (μS)	PIC (*n* = 41)	65.8 ± 5.3 [55.0, 76.6]	102.5 ± 8.2 [85.9, 119.0]	36.7 ± 6.3	24.9 ± 7.7	0.001	0.715
	Placebo (*n* = 41)	63.0 ± 5.5 [51.9, 74.2]	74.7 ± 5.9 [62.8, 86.7]	11.7 ± 4.4			
Corneometer (AU)	PIC (*n* = 41)	31.0 ± 1.4 [28.0, 33.9]	39.5 ± 1.6 [36.4, 42.7]	8.6 ± 1.5	4.3 ± 2.4	0.044	0.395
	Placebo (*n* = 41)	31.1 ± 1.3 [28.5, 33.8]	35.4 ± 1.5 [32.5, 38.3]	4.3 ± 1.9			

μS, microsiemens; AU, arbitrary units; PIC, piceatannol; SE, standard error; *p*-values indicate between-group differences calculated by analysis of covariance (ANCOVA), with baseline values as covariates.

### 3.3 Wrinkles

After 8 weeks of intake, the PIC group showed a significantly greater reduction in wrinkle grades than the placebo group (*p* = 0.009), indicating an improvement in crow’s feet. To visually illustrate the effects of PIC intake, graphical representations of wrinkle grades are provided in the Supplementary Figure S2. Table 3 presents the wrinkle grades.

**Table 3:** Measurement of wrinkle grade.

Evaluation parameter	Group	Baseline	Week 8	Change from baseline	Group differences	*p*-value	Cohen’s *d*
		Mean ± SE [95% CI]	Mean ± SE	Mean ± SE	Mean ± SE		
Wrinkle grades	PIC (*n* = 41)	2.92 ± 0.12 [2.68, 3.16]	2.83 ± 0.12 [2.59, 3.08]	−0.088 ± 0.020	−0.076 ± 0.028	0.009	0.609
	Placebo (*n* = 41)	3.05 ± 0.13 [2.80, 3.31]	3.04 ± 0.13 [2.78, 3.30]	−0.012 ± 0.019			

PIC, piceatannol; SE, standard error; *p*-values indicate between-group differences calculated by analysis of covariance (ANCOVA), with baseline values as covariates.

### 3.4 Safety evaluation

During the food-intake study period, one adverse event was reported in a participant in the PIC group. The event was mild, with no serious adversity. The reported event was transient urticaria, which the study physician considered to be unrelated to the test food.

## 4 Discussion

In this study, an 8-week intervention with a PIC-containing beverage resulted in a significant increase in stratum corneum hydration in the PIC group than in the placebo. Furthermore, visual assessment of wrinkle grade showed a significant improvement in the PIC group. The findings suggested that PIC, as a dietary ingredient, is effective not only for enhancing skin moisture retention but also for improving age-related wrinkle formation.

Till date, several human studies have demonstrated that PIC ingestion can improve stratum corneum hydration and skin elasticity (24). However, this is the first clinical trial to concurrently evaluate its pronounced effects on both stratum corneum moisture retention and wrinkle improvement. Although other food-derived ingredients—such as bilberry extract (28) and bonito elastin–derived peptides (29) —have also been reported to alleviate wrinkles, the total number of clinical investigations remains small. While direct head-to-head comparisons of PIC with these or other materials have not yet been performed, PIC might represent a promising dietary ingredient for enhancing skin hydration and mitigating age-related wrinkle formation.

Possible mechanisms underlying the moisture-retention effect of PIC include its regulation of both the synthesis and degradation of key dermal matrix components, namely collagen and hyaluronic acid. In human dermal fibroblasts, PIC has been shown to increase collagen production (30) while inhibiting the expression of matrix metalloproteinases (MMPs), the principal enzymes responsible for collagen breakdown (31). Further, it upregulates hyaluronan synthases and suppresses hyaluronidases (32, 33), thereby promoting hyaluronic acid accumulation in the skin. The actions are mediated, at least in part, by SIRT1, a longevity-related deacetylase; enhanced SIRT1 expression has been reported to stimulate the transcription of collagen-synthesizing enzymes (34, 35) and repress MMP expression (36). Studies using SIRT1 inhibitors have demonstrated that PIC induces the gene expression of HAS2—the key hyaluronic acid synthase—in a SIRT1-dependent manner (32). Moreover, PIC itself has been shown to elevate SIRT1 gene expression in skin fibroblasts (32). Together, PIC has been reported to help maintain dermal collagen and hyaluronic acid levels through SIRT1 activation, thereby preserving dermal structure and enhancing the dermis’ water-holding capacity and supply moisture to stratum corneum. Improved hydration, in turn, could alleviate wrinkle formation, since reduced stratum corneum water content is associated with increased skin stiffness and deeper, more extensive wrinkles (37). Our clinical results were consistent with this mechanism, indicating that the moisture-retaining action of PIC likely contributes to the observed improvement in dryness-induced wrinkles.

In this study, we did not observe a clear correlation between an increase in stratum corneum hydration and improvements in wrinkle grade, suggesting that the anti-wrinkle effects of PIC might involve mechanisms beyond enhanced moisture retention. One plausible pathway is the upregulation of SIRT1, leading to antioxidant and anti-inflammatory actions. Wrinkle formation is known to be driven not only by skin dryness but also by inflammation, degeneration of dermal collagen fibers, and oxidative stress (38, 39), and SIRT1 has been implicated in the regulation of these processes (40, 41). Indeed, PIC has been reported to exert both antioxidant and anti-inflammatory effects in skin-derived cells and tissues (21, 42, 43), and such bioactivities might contribute to its ability to improve wrinkle appearance.

The current study demonstrated improvements in stratum corneum hydration using two different measurement devices, namely the SKICON-200EX and the Corneometer. The SKICON-200EX employs a high-frequency conductance method that is considered to be particularly sensitive to moisture in the more superficial layers of the stratum corneum, whereas the Corneometer uses a capacitance technique to reflect hydration across the entire thickness of the stratum corneum. The fact that both instruments—despite their differing measurement principles—consistently detected increased water content suggested that PIC enhanced hydration throughout the stratum corneum, regardless of the measurement depth.

The improvements in stratum corneum hydration and wrinkle appearance observed in this study have implications that extend beyond esthetic benefits and may contribute to the extension of healthy life expectancy in older adults. The World Health Organization has recently reframed healthy aging under the concept of “Intrinsic Capacity” which comprises five interrelated domains, namely sensory function, locomotor capacity, cognition, psychological well-being, and vitality (44). Among these, skin health—particularly parameters such as hydration and wrinkle severity—has been shown to influence psychological aspects, including self-esteem and motivation for social engagement, as well as one’s overall sense of vigor (6). By enhancing the skin’s barrier function and improving visible signs of aging, PIC might support the psychological and vitality domains of Intrinsic Capacity, eventually helping to maintain or even enhance overall functional capacity in the aging population.

Our current study has several limitations. Potential confounding factors, such as the participants’ age range and gender, could not be fully controlled, and the scope of the evaluation metrics was limited. In addition, the evaluated endpoints were limited to skin hydration and wrinkle severity; moreover, wrinkle assessment was based solely on visual grading, and objective instrumental analyses were not performed. Further validation using instrumental measurements is warranted in future studies.

Furthermore, since we screened candidates based on low stratum corneum hydration, our findings may not be generalizable to individuals with normal or higher hydration levels. Finally, the durability of the observed improvements in wrinkle severity and stratum corneum hydration, as well as a detailed understanding of the underlying mechanisms, would require further investigation in future studies.

Future studies should broaden the diversity of study populations, explore different intervention durations and dosages, and investigate the molecular pathways underlying PIC’s actions as well as its effects on additional skin-function endpoints—such as elasticity, colour tone, and barrier recovery. Such comprehensive, multidimensional, and application-oriented research will be essential for fully characterizing the utility of PIC as an internal skin-health ingredient.

## 5 Conclusion

In healthy adult women, daily intake of PIC for 8 weeks led to a statistically significant increase in stratum corneum hydration and improvement in wrinkle severity than the placebo group. The findings suggest that PIC may be a valuable dietary ingredient for enhancing skin moisture retention, and improving the appearance of crow’s feet. PIC may also help suppress wrinkle formation.

## Data Availability

The datasets presented in this article are not readily available due to privacy concerns. Requests to access the datasets should be directed to SK, s-kawakami-jf@morinaga.co.jp.
