# Supplementary material for: Oral supplementation with piceatannol improves skin hydration and reduces wrinkle severity: a randomized, double-blind, placebo-controlled trial
Source: Front Nutr. 2026 Apr 28;13:1765478. doi: 10.3389/fnut.2026.1765478 (PMC13163207; doi:10.3389/fnut.2026.1765478)
Supplement: Supplementary file 1 [file Image_1.pdf]

Supplementary Material

1 Supplementary Figures and Tables

1.1 Supplementary Figures

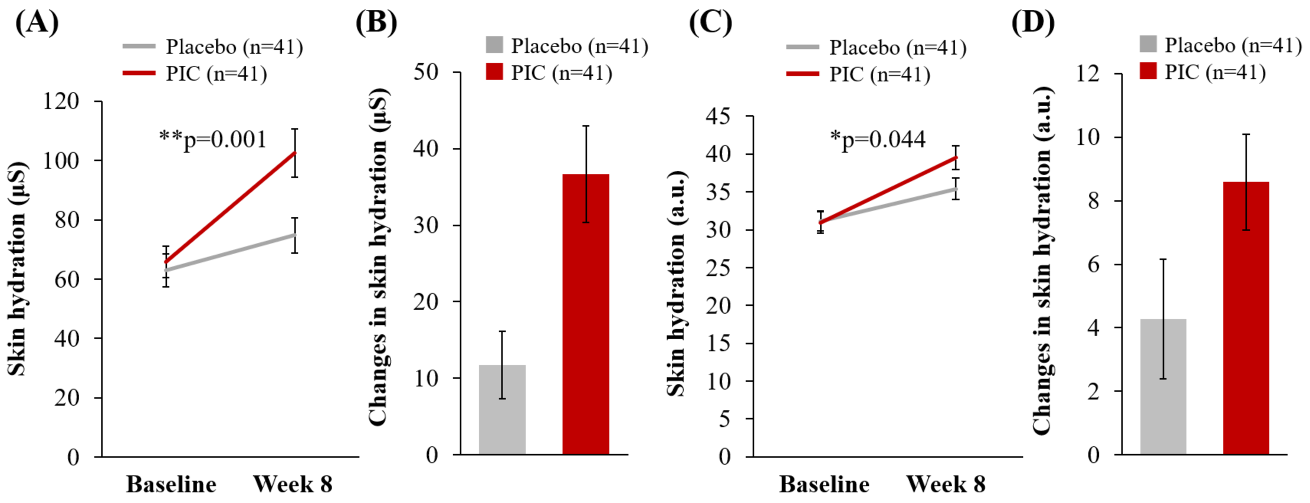

Supplementary Figure 1 Changes in skin hydration following intake of piceatannol (PIC) or placebo

Stratum corneum hydration measured using the SKICON-200EX at (A) baseline and after 8 weeks of intake (week 8), and (B) as changes from baseline to week 8. Hydration was also measured using the Corneometer CM825 at (C) baseline, and week 8, and (D) as changes from baseline to week 8. Data are presented as mean ± standard error. p values indicate between-group differences calculated by analysis of covariance (ANCOVA) with baseline values included as covariates.

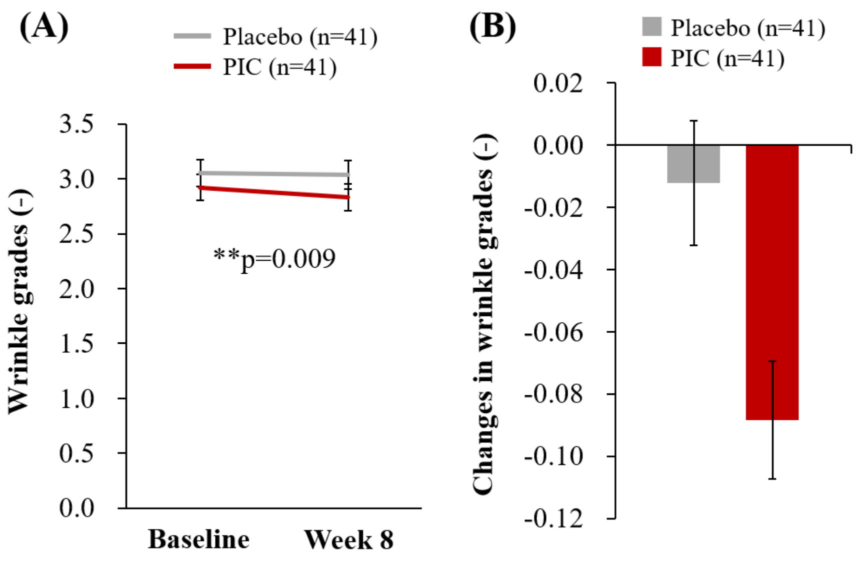

Supplementary Figure 2 Changes in wrinkle scores following intake of piceatannol (PIC) or placebo

(A) Wrinkle scores at baseline, and week 8. (B) Changes in Wrinkle scores from baseline to week 8. Wrinkle severity was visually evaluated according to the guidelines of the Japanese Cosmetic Science Society (JCSS). Data are presented as mean ± standard error. p values indicate between-group differences calculated by analysis of covariance (ANCOVA) with baseline values included as covariates.
